# The Effect of Patient Age on Standardized, Uptake Value-Hounsfield Unit Values of Male Genitourinery Structures In F-18 FDG PET/CT

**DOI:** 10.4274/MIRT.40

**Published:** 2011-12-01

**Authors:** Berrin Çavuşoğlu, Hatice Durak

**Affiliations:** 1 Dokuz Eylül University The Institute of Health Sciences Department of Medical Physics, Izmir, Turkey; 2 Dokuz Eylül University Medical School Department of Nuclear Medicine, İzmir, Turkey

**Keywords:** PET/CT, SUV, HU, genitourinary system

## Abstract

**Objective:** Relation between patient age and Hounsfield Unit (HU),which is the linear attenuation coefficient, and Standardized Uptake Values (SUV) which is the amount of 18F-fluorodeoxyglucose (F-18 FDG) uptake, measured in the areas of interest drawn to prostate, seminal vesicles and testicles in F-18 FDG Positron Emission Tomography/Computed Tomography (PET/CT) images was investigated.

**Material and Methods:** Mean and maximum SUV and HU values were recorded from the areas of interest (min 12 mm in diameter) which showed FDG uptake in prostate, seminal vesicles and testicles from F-18 FDG PET-CT images of 21 male patients under 40 years without genitourinary cancer. The effect of patient age to SUV and HU values was examined with Pearson correlation test using SPSS program.

**Results:** There was a negative insignificant correlation between patient age and SUV and HU values for prostate. For seminal vesicles, correlation between patient age and SUV values and HU_max _were positive but insignificant, while correlation with HU_mean_ was significant (r=0.459, p=0.00). Correlation between patient age and SUV_max_ and SUV_mean_ values were significant for testicles (r=0.506, p=0.002 and r=0.467, p=0.005, respectively) but the correlation between patient age and HU_max _and HU_mean_ values was not significant.

**Conclusion:** F-18 FDG uptake in testicles in males increases with age until 40, suggesting an increase in metabolic rate. The significant correlation between age and mean HU values is probably caused by thickening of the tissue without an increase in glucose metabolism in seminal vesicles. In prostate, the effect of patient age to SUV and HU values was not observed until the age 40.

**Conflict of interest:**None declared.

## INTRODUCTION

Integrated Positron Emission Tomography/Computed Tomography (PET/CT) allows morphological and functional imaging in a single imaging process by combining PET and CT in a single device and is an important diagnostic imaging tool for the identification, localization and characterization of different group of malignancies (1).

Determination of the characteristics of the lesions can be made by examining the Standardized Uptake Value (SUV) and Hounsfield Unit (HU) values in integrated PET/CT systems. It is important to know the value of the normal tissues for appropriate evaluation of pathology. Uptake of F 18 Fluorodeoxyglucose (F-18 FDG) is frequently observed in varying degrees in the male genitourinary structures and sometimes physiological uptake is confused with pathology. Physiological uptake and also the tissue density in genitourinary structures may be expected to change with age, associated to metabolic and histological changes due to maturing and aging. (2) We aimed to investigate the relation between patient age and Hounsfield Unit (HU) and SUV values measured in the areas of interest drawn to prostate, seminal vesicles and testicles, where sometimes high uptake is observed in F- 18 FDG PET/CT images.

## MATERIALS AND METHODS

Twenty two male patients under 40 years of age who has undergone F-18 FDG PET/CT without genitourinary cancer or any previously reported abnormal findings in the genitourinary system were selected from the database. The age range was 5-37 and the mean age was 25.0±8.1. In all patients, PET/CT was performed one hour after the injection of 7 - 15 mCi F-18 FDG using Philips Gemini TF 16. The patients fasted at least 4 h prior to intravenous injection of F-18. No patients had diabetes or blood glucose levels over 140 mg/dL PET was performed as 1.5 minutes per bed position from the base of the head to the mid thigh. CT parameters were 50- 80 mAs 120 kVp, 5 mm slices at 39 mm/sec in 512 x 512 matrix. Primary diagnosis of patients were as follows: NonHodgkin's lymphoma (n=2), osteosarcoma (n=1), lymphoblastic lymphoma (n=1), sarcomatoid carcinoma (n=1), mixed germ cell tumor (n=1), germ cell tumor (n=1), anaplastic large cell lymphoma (n=2), leimyosarcoma (n=1), Hodgkin's lymphoma (n=3), metastatic tumor (n=1), hamartoma (n=1), paraneoplastic syndrome (n=1), soft tissue sarcoma (n=1), stomach cancer (n=1), malign mesenchymal tumor (n=1), Kaposi's sarcoma (n=1) and diffuse large B cell lymphoma (n=1).

**Figure 1:**
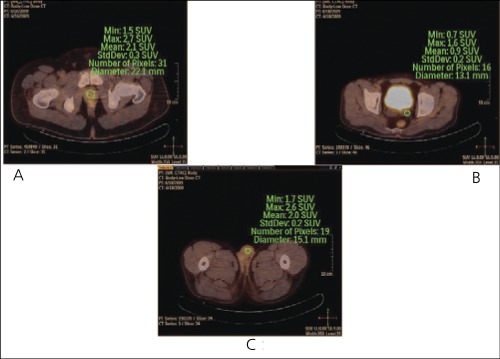
Regions of Interests drawn on prostate (A), seminal vesicles(B) and testicles (C)

SUV and HU values were recorded from the areas, which showed FDG uptake in prostate, seminal vesicles and testicles using the transaxial slices of fused F-18 FDG PET-CT images (Figure 1). Maximum and mean SUV and HU values from the regions of interests with a minimum of 12 mm in diameter were obtained (3). If one of the seminal vesicles or testicles were not visualized enough to place a ROI, SUV and HU values were taken from only one side in some patients (for seminal vesicles bilateral in 19 patients, unilateral 2 in patients; for testicles bilateral in 12 patients, unilateral in 10 patients).

Mean and standard deviation values were calculated for maximum and mean SUV and HU values. The effect of patient age on SUV and HU values was assessed with Pearson correlation test using SPSS program. p<0.05 was considered significant.

## RESULTS

Mean patient age, mean SUV_max_ and SUV_mean_, mean HU_max_ and HU_mean_ values obtained from the areas of interest are shown in the Table 1. Though we did not perform any statistical analysis regarding the SUV and HU values to compare the organs, SUVmax was higher in seminal vesicles compared to testicles and prostate, but there was a wide standard deviation. Both HUmax and HUmean were higher in prostate gland compared to testicles and seminal vesicles. Correlations between patient age and SUV_max_, SUV_mean_, HU_max_, HU_mean_ values are shown in Table 2.

**Figure 2:**
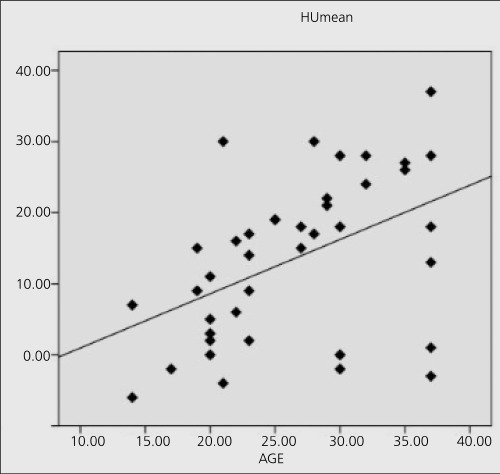
Distribution of HUmean values according to patient age inseminal vesicles and the linear fit curve (p=0.003)

**Figure 3:**
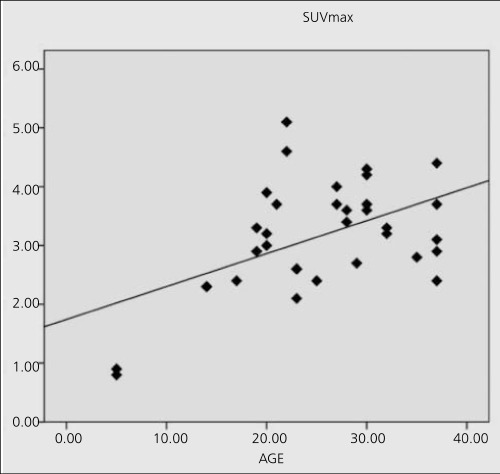
Distribution of SUVmax values according to patient age intesticles and the linear fit curve (p=0.002)

There was a negative insignificant correlation between patient age and SUV and HU values for prostate. For seminal vesicles, correlation between patient age andHUmean was significant (r=0.459, p=0.00) (Figure 2). Correlation between patient age and SUVmax and SUVmean values were significant for testicles (r=0.506, p=0.002 and r=0.467, p=0.005, respectively) (Figure 3)

## DISCUSSION

Our patient selection involved patients from puberty to up to age 40 to reveal the effect of maturation of the organs on metabolic and tissue density parameters. Therefore, we did not include the effect of advanced age, which may be also studied by others as long as there are normal patients available in patient database. Occult prostate carcinoma may be present in the elderly and it is not possible to evaluate any 18F FDG uptake as physiologic or pathologic unless a carcinoma is excluded by biopsy (4). In prostate gland, we did not observe any significant effect of patient age to SUV and HU values. It seems that the metabolic activity and tissue density of the prostate gland do not significantly change from puberty to middle age. Jadvar et al (5) also reached this conclusion. They concluded their aforementioned study stating that the normal prostate size increases with age, it does not significantly affect metabolism of the gland and CT density.

The significant correlation between age and mean HU values in seminal vesicles is probably caused by thickening of the tissue without an increase in glucose metabolism in seminal vesicles. In Magnetic Resonance TI weighted images, normal seminal vesicle signal intensity was shown to be similar to that of muscle or bladder. Dihydrotestosterone and testosterone propionate are shown to increase the weight and the collagen content of the seminal vesicles in rats (6). Our group consists of men between ages 14 and 37, so development of the seminal vesicles may be reflected by the increase in HU values.

We found that F-18 FDG uptake in testicles in males increased significantly with age until 40 suggesting an increase in the glucose metabolic rate. A strong positive correlation between F-18 FDG uptake in the testis and age was shown in pediatric age group, between 9 to 17 years of age. The correlation coefficient was found as 0.406 in 42 PET-CT studies (7). Actually, the curve in Figure 3 may be biexponential, showing a fast increase in SUV_max_ in pediatric ages followed by a mild slope after adolescence which was similarly reported in the literature that testicular metabolic activity demonstrated an increasing trend until the age of 35 with a plateau afterwards until the age of 65 years (8). An increase in testicular volume with age from birth to 25 years was reported. After age 25, testicular volume started to decline and testicular volume was positively correlated with age (r=0.67) (8). According to Yang et al (9) testicular volume rapidly increases during puberty and peaks at age 30 years. Though we had a few pediatric patients, we have seen a significant correlation. We did not calculate the testicular volumes, but it seems that partial volume may have an influence on the SUV values causing an overestimation of the correlation with age. Kitajima K et al reported the SUV value as 2.81±0.43 between the ages 30-39 years (10). Our mean SUV_max_ (3.2±0.9) was a little bit higher perhaps due to the younger age of the patients. It has been reported by Amrolia et al. (11) that Leydig cells which produce testosterone take up glucose by a transport system that is similar to the facilitated-diffusion system of glucose uptake in other mammalian cells. Therefore, increased FDG uptake in the testes of the younger man and reduction in elderly may be caused by the changing of the number and production ability of Leydig cells (11).

In conclusion, we observed that F-18 FDG uptake in testicles in male increases with age until 40 suggesting an increase in metabolic rate. It seems that in seminal vesicles, there is an increase in tissue density without an increase in glucose metabolism. In prostate, the effect of patient age to SUV and HU values was not observed until the age 40.

## Figures and Tables

**Table 1:**
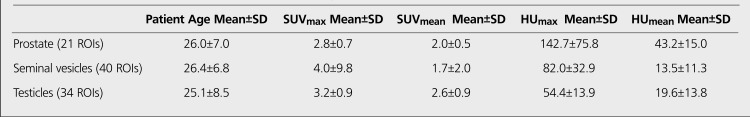
The standard deviation and mean values of patient age, SUV_max_, SUV_mean_, HU_max_ and HU_mean_ in different structures

**Table 2:**
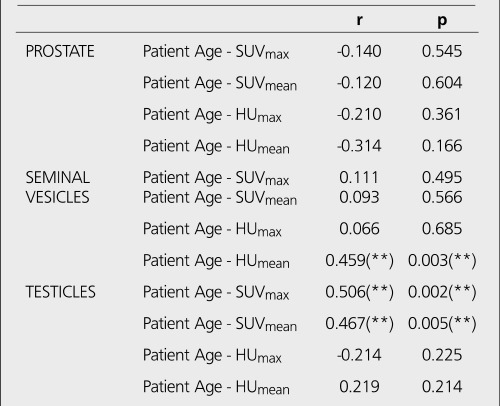
Correlation analysis between patient age and SUV_max_,SUV_mean_, HU_max_ and HU_mean_ values
